# The biofabrication of ZnO nanoparticles using the green soft technique reduction of Zincum Gluconicum (ZNG) by extracellular mycofiltrate of *Penicillium italicum* Pit-L6

**DOI:** 10.25122/jml-2021-0327

**Published:** 2022-12

**Authors:** Mohammed Abdul-Sahib Issa, Zaman Kareem Hanan

**Affiliations:** 1 Department of Biology, College of Science, University of Thi-Qar, Nassiryhia, Iraq; 2 College of Dentistry, University of Thi-Qar, Nassiryhia, Iraq

**Keywords:** ZnONps, zincum gluconic, *Penicillium italicum* Pit-L6, SEM, TEM

## Abstract

Recently, biological techniques for manufacturing nanoparticles, such as employing filamentous fungi to synthesize ZnO nanoparticles, have become environmentally friendly, bio congruous, and safe. This study aimed to look for *Penicillium italicum* [Filamentous Blue Mold (FiBM)] in rotting citrus fruits and exploit this in the biofabrication of ZnO nanoparticles. The study isolated 39 different filamentous mold samples and used conventional and molecular diagnosis. Only 11 (28%) of the isolates obtained contained *Penicillium italicum*, for which we investigated the capability of ZnO nanoparticles biosynthesis by fungal extracellular free-cells filtrate solution. The results showed that *Penicillium italicum* Pit-L6 was given the peak of ZnONps 378 nm detected by UV-visible spectrophotometry, and it considered significantly optimum strain in the highest quantity (mean±S.D) 0.015±0.002 gm/100 ml with small enough average nanoparticles size. The ZnONps were characterized by UV-visible scanning spectrophotometry, atomic force microscopy (AFM), X|-RD, scanning electron microscopy (SEM), and transmission electron microscopy (TEM). The final average ZnONps through 0f in all measuring devices ranged between 53.13–69.67 nm (with different shapes and dimensions). This study concluded that these fungi (FiBMs) are highly capable as eco-friendly and cheap bio-nano factories to manufacture ZnONps as alternative novel biological technology, in fine particles within average size at nano-level, as continuous renewable sources for producing nanoparticles, for different usage.

## INTRODUCTION

Recently, it was discovered that enabling metal nanoparticles (MeNps) with extremely small particle sizes, preferably less than 100 nm, gives unique characteristics to this novel concept. Because of their incredibly small size and high surface area to volume ratio, MeNps exhibit distinct characteristics compared to their bulk counterparts. In this respect, Nps have been widely employed in photonics, microelectronics, information storage, catalyzis, energy conversion, and communications [1, 2]. Furthermore, these are used in medical applications and pharmaceuticals, such as the Newcastle disease vaccine, using gold nanoparticles [3]. Tuhmaz (2020) studied the improvement of thermo-optical cancer treatment by laser light with Ag nanoparticles [4] and other applicable fields.

Zinc oxide nanoparticles (ZnONps) have gained considerable popularity in recent years due to their ability to cause unusual characteristics in semi-conductors by submitting an elevated gab of band with 3.4eV in addition to the binding energy of 60meV, resulting in peculiar electrical and optical properties [5]. Zinc, as a metal, is a vital component of living creatures. As a result, both prokaryotes and eukaryotes, such as bacteria, fungi, and yeast, produce ZnONps using microbial cells or enzymes, proteins, and other biomolecules as microfactories. Although ZnONps have antibacterial capabilities, nanoparticles (Nps) have qualities that depend on their size and form, making them specialized for different purposes. NPs are distinguished by size and form, ideal for various applications [1]. Alavi and Nokhodchi (2020) suggested the potential of ZnONPs combined with biopolymers like cellulose, chitosan, and alginate as an alternative way to increase the mechanical and antibacterial properties of wound-healing tissue scaffolds [6]. Additionally, Alavi and Nokhodchi (2021) revealed that compared to Gram-positive bacteria, the protein leakage and DNA avalanching through loose membranes are more affected than those of Gram-negative bacteria due to the action of Ag and ZnONPs, making it possible to use these particles as antibacterial agents similar to antibiotics [7].

Nanoparticles were created using biological techniques and with the help of biologically energetic molecules, including bacteria, fungi, plants, and yeasts. Because of their immobility, greater metal bioaccumulation capability, and high binding efficiency, fungi were preferred over bacteria in most cases. Fungi have a wide range of uses due to their ability to generate vast amounts of enzymes, ease of scale-up, low cost, and ease of managing biomass [8]. Hefny *et al*.(2019) used culture filtrates from several species of *Aspergillus, Fusarium*, and Penicillium synthesizing ZnONps [9], while Issa *et al*.(2018) [10] used *Aspergillus parasaticus* Ap4 and Housseiny and Gomaa (2019) [11] employed *Penicillium chrysogenum* AUMC 10608 for the same purpose.

*Penicillium italicum*, often known as filamentous blue mold (FiBM), is a major source of postharvest citrus rot with a wide range of intra-extracellular catalyoreducing enzymes. Therefore, this study was the first to investigate the efficacy of *P. italicum* filtrate in reducing zincum glucocnicum hydrate in biomanufacturing ZnONps as a simplified, cost-free, and eco-friendly route and to support the bio fabrication trajectories of other nanoparticles serving various scientific, medical, and commercial needs.

## MATERIAL AND METHODS

### Chemicals

The analytical grade of all the chemicals and reagents listed below was used in this study: Zincum Gluconicum (ZnG) hydrate (C_12_H_22_O_14_Zn) (Analar_ England), PDagar and PDbroth, malt extract, peptone, and agar (Himedia_ India), glucose, NaOH, HCl, and chloramphenicol (BDH _ England), KH2PO4 and K2HPO4 (CDH _India), MgSO4.7H_2_O, and (NH4)2SO4 (Merek_Germany).

### FiBMs isolation

All citrus fruits in this project were obtained at a local market with decaying morphology: *Citrus amarra, Citrus aurantium, Citrus limone, Citrus maximae*, and *Citrus reticulate*. Afterward, the surface was sanitized with cotton soaked in 70% ethanol. Following this, the fruits were chopped into small pieces (1.5–1.5 cm) with a sterile scalpel. Infected sample segments were then plated in threes on solidified PDA (10 gm/250 ml) treated with chloramphenicol (25 mg/50 ml) [12, 13]. Inoculated plates were incubated at 28±1℃ for five-seven days. Several fungal isolates with varying colorations were observed from the incubated plates, including; "i" Green or Blue-Grey' which indicated that the target was in the Penicillium genus. The fungal colonies formed were periodically subcultured to create a pure culture of the fungal isolates (a sterile sharp pointed needle was used to pick off the edge of a growing colony). The pure isolated fungus was kept on the PDA slant and stored in the fridge at 4℃ for further investigation [14].

### Identification of pure FiBM strains

The same pure isolated fungus was detected and diagnosed simultaneously at the Central Fungi Laboratory, Faculty of Science, University of Basra, following the most well-documented and protocol-based keys to fungi identification [15, 16]. The FiBM isolates were submitted to morphological and microscopical examinations. The molecular PCR technique proved the diagnosis using Quick Genomic DNA Extraction Kit [N1111/N1112-(GDSBio-China)] for DNA fungal extraction, one and a half milliliter of 18–20 hour incubated growth culture of PDB centrifuged in 1.5 ml Eppendorf tube at 12.000×g for five minutes. Then, the supernatant and the remaining pellet were employed for total DNA extraction following the manufacturer's recommendation, the specific primers (RPB1-a) shown in Table 1, and the thermal program of PCR conditions for *P. italicum* detection [17]. The PCR reaction mixture was 25 µL in a total volume containing 12.5 µL of premixed ready-to-use solution master mix (2X Taq Mix- Dongsheng Biotech, China), which consisted of 0.25U/ul Taq DNA polymerase, 2X PCR buffer, 0.4 mM dNTPs, 3.2 mM MgCl_2_, 0.02% bromophenol blue, 1 µL of retrieved genomic DNA (10pmol to1µL), both forward and reversed primers, and 9.5µL of nuclease-free double distilled water (ddH_2_O). GradientVeritti TM96-Well Thermal Cycler (AB~Applied Biosystems-Singapore) thermo-controller was used for amplification. All fungal strains were kept in two duplicates on a slant (universal glass tube) on "MGPA" made up of 2% g/l malt extract, 2% g/l glucose, 0.1% g/l peptone, and 1.5% g/l agar in the form of stock cultures at 4–8°C. Subculturing was used to revive these cells regularly [18].

**Table 1:** The specific primers and PCR conditions for detecting FiBM isolates.

Primer code	PCR sequence (5'→3')	Annealing temp (°C)	Goal	Amplicon size (bp)	Target gene
**RPB1-a -F**	TGCGGTATCTACAAGATT-18b	64	*Penicillium italicum* detection	790	RPB1- [RNA polymerase II largest subunit]
**RPB1-a -R**	AGTGAGGAAGAGTACGAT-18b				

### FiBM biomass and active mycofiltrate preparation

According to a study by Issa *et al*. (2018) [10], one batch of rocking aerobically submerged fermentation (bra-Smf) in 250 ml Erlenmeyer flasks (Em.f) was used to produce FGM biomass in 100 ml base fortified malt extract peptone glucose (FMEPG) liquid media at specific incubation parameters, until the reducing active broth was obtained, which was then used for the big originate of ZnO nanoparticles.

### Zincum gluconicum hydraite reducing to ZnONps by mycofiltrate

About 50 ml of one-and-a-half millimole Zincum Gluconicum hydrate was dissolved in 341.775 mg C_12_H_22_O_14_Zn in 0.5 L ions-free autoclaved water (i.f.a.w) in an Em.f of 250 ml. The final concentricity solution was blended with 50 ml of activation fungal filtrate, and pH was adjusted to 7.0, with two glasses flasks of 250 ml, one containing mycofiltrate (without C_12_H_22_O_14_Zn) and the other containing just pure Zincum Gluconicum solution (without mycofiltrate) as positive and negative controls. Each isolate was cultured in an orbital shaking incubator with 145 jerk/min at 30℃ for 3 days in triplicate [10]. When the white precipitate began to develop at the bottom of the flask, the transformation process was completed, creating ZnONPs, which helped determine which strains were effective in producing ZnOnps. The accumulation was then extensively washed with i.f.a.w to eliminate all ions and other contaminants before filtering using Whatman (No.1) paper and a 0.45 µm Millipore filter under vacuum pressure in reverse to increase the purity [19]. The white aggregate formed at the flask base was separated from the filtrate (15 ml centrifuge tubes) using centrifugation twice at 10,000 xg for 15 minutes with a 5-min pause. Finally, a vacuum oven at 105℃ was used to dry the milky-white precipitate. The fine powder of ZnONPs extracted was then weighed in preparation for the subsequent investigations [20].

### Election of the superior strain in the manufacture of ZnONps

The selection of the best fungal strains capable of fabricating ZnONPs at the quantity and quality levels set was based on three factors: the ability to create foggily and turbidity in a bioreactor reaction of mycelial aqueous extract in the early stages. Second, a portion (2 ml) of the hazy solution was subjected to UV-Vis. spectral analysis using a scanning UV-spectrophotometer (UV-1800-Shimadzu-Japan) with spectrum values obtained in the wavelength range of 300–600 nm at various time intervals (24, 48, and 72 hrs). The ZnONps were between 340–390 nm wavelength according to standard norms. Finally, the formation of white precipitate at the bottom of the bioreactor flask proved that the fungal strain could generate ZnO nanoparticles quantitatively [14]. The tiniest particles in diameter and average size, on the other hand, were qualitatively screened using an Atomic Force Microscope (AFM) with Scanning Probe Microscopy (SPM) (Nanocompact AFM/PHYWE – Germany), with Silicon nitride cantilever, and pyramidal tips were used for the analysis of the samples. The cantilever was set at a resonating frequency of 70 kHz with a length of 115 µm with a spring constant (stiffness) of 0.5 N/m. The best isolates for further research in the nanoparticle manufacturing industry are chosen based on these two factors [21].

### Characterization of finished ZnO nanoparticles

#### X-ray diffraction

The study looked into particle size, crystal structure, and surface morphology during the structural characterization. X-ray diffraction is a technique used to study objects' structure which was used at the Central Laboratory in the Ibn Al-Haithiam of the Education for Pure Sciences Faculty, University of Baghdad, Iraq, using a diffractometer recorded in the range of 20≤2ɵ≤90 angles and using Cu- Kα as an anode. The Debye-Scherrer equation may be used to determine the average crystallite size [22].


$$D_{hkl}=k\times \lambda /\beta_{hkl}\times cos\;\theta_{hkl}\;\;\left(1\right)$$

Where: D_hkl_=is the ordered (crystalline) domains of the mean size, hkl=are the planes being analyzed of the Miller indices, K=0.88~0.9 is the Schirrer equation, λ=the wavelength of X-ray, β=the maximum intensity of the line broadening at half [FWHM], θ=the Bragg angle.

#### SEM and TEM imagination studies

The fabricated and annealed surfaces of ZnO nanopowders were scanned via electronic scanning microscopy (SEM112544/Angistrom Advanced-USA) in the Materials Research Det. SEM-Unit of Materials Research Det. at the Ministry of Science and Technology to capture micrograph pictures of manufactured ZnONPs and study the nanopowder's homogeneity. Also, the architectural morphology and dimensional nanostructures (average particles size) of ZnONPs powders were measured and confirmed, which were manufactured by the different FiGM isolates through the transmission electron microscope (TEM)(CM10/Philips- Holland) measurements (average size) provided with carbon-coated copper specimen holder grids. The TEM micrographs were obtained in the Technograph Unit of TEM at the Faculty of Medicine, Al-Nahrain University/Iraq, at a low voltage of 100 kV.

### Statistically outcome analysis

In all trials, the mean of the findings, standard deviation or error (SD or SE), respectively, of the triplicate of each isolate were calculated. IBM SPSS software (V.22) was used for statistical analysis. One-Way-ANOVA and Post-hoc-Tukey/ALPHA=0.01 tests were used to discover differences between means. A p-value of <0.01 was considered significant.

## RESULTS

### Identification of FiBMs isolates

The collection effort was focused on 11 of 39 isolates of *Penicillium italicum* from various citrus fruits, as shown in Table 2.

**Table 2:** Outcome and numbers of *P. italicum* isolates recovered from different citrus fruits.

Isolate code	Source of isolation	Total
**Pit-M1**	*Citrus reticulate* (Mandarin)	3
**Pit-M2**		
**Pit-M3**		
**Pit-B4**	*Citrus amara* (Bitter orange)	1
**Pit-P5**	*Citrus maxima* (Pomelo)	1
**Pit-L6**	*Citrus limon* (Lemon)	3
**Pit-L7**		
**Pit-L8**		
**Pit-O9**	*Citrus aurantium* (Orange)	3
**Pit-O10**		
**Pit-O11**		

**Figure 1:**
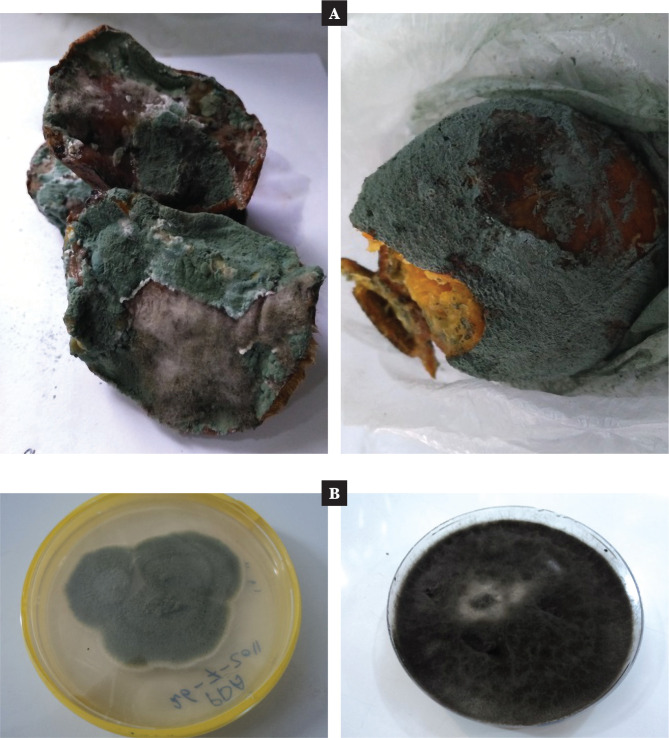
A – the assortment of rotten citrus as sources of the FiBms isolates; B – pure culture belonging to different FiBms isolates.

The study deliberately targeted isolates of the fungus *Penicillium italicum* (Blue-olive rot) from citrus fruits shown in Figure 1 A and B. The diagnosis was made on many features, including the phenotype (morphological and microscopically) based on specific classification keys during the first ten days of February 2021, finally confirmed by molecular technique via conventional PCR. This result corresponded to Al-Zamily *et al*. (2016) [23], who isolated different fungal genus from citrus fruits to produce single-cell oils.

**Figure 2:**
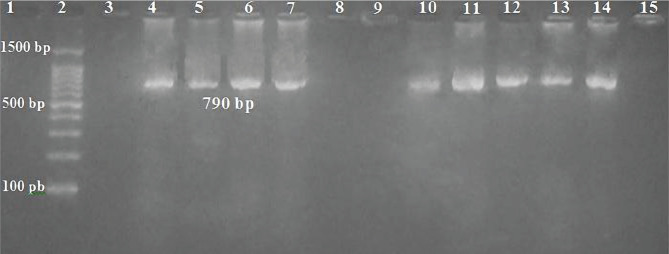
Gel electrophoresis amplified PCR product of RPB1gene (790 bp) in monoplex PCR at 70 v for 90 min in 1.7% agarose, TBE (1x), stained with ethidium bromide. Lane (2): DNA ladder (100 bp), lane (1) negative control (without DNA), all the lanes were positive for the target gene except lanes (3), (8), (9), and (15) which were negative results (belonged to other species).

The results of the genotypic diagnosis are shown in Figure 2. Only eleven (28%) isolates were confirmed as *Penicillium italicum* of 39 isolates, which were subjected to the biosynthesis screening of ZnO nanoparticles. The specific primers RPB1-a [RNA polymerase II largest subunit] used to detect *P. italicum* rapidly significantly facilitated the diagnostic method among a large number of unknown citrus postharvest pathogens, especially filamentous fungi, which match the results of Chen *et al*. (2019) [17].

### Fabrication and characterization of Myco-ZnONps by mycofiltrate of FiBMs

**Figure 3:**
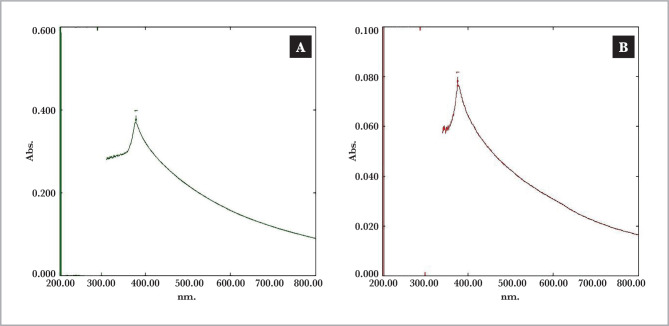
UV-visible peak plots in firstly election of *Penicillium italicum* Pit-L6 (A), Pit-B4 (B).

Table 3 contains the results of all *penicillium italicum* isolates that were given suitable peaks in the ZnONps 320~390 nm range. However, only five isolates (Pit-M2, Pit-B4, Pit-L6, Pit-L7, and Pit-O9) created the misty state in the filtrate reaction, resulting in the white-milky nanopowder deposited, as shown in Figure 3 A and B. Some strains received ZnONps peak but could not produce the misty form and instead deposited white powder as in Pit-M1, Pit-L8, and Pit-O11. Others were given a foggy white hue but could not precipitate nanopowder, such as Pit-M3, PitP5, and Pit-O10, which cannot produce ZnONps.

**Table 3:** The results of UV. visible peaks, mean sum of nanopowder weight, and mean size Nps by AFM of *P. digitatum* isolates capable of ZnONps bio-fabrication.

FiGM strains	Mean±S.D of UV-visible position peak (nm)	Mistiness formation	Presence of white precipitate	Mean sum of nanopowder weight (gm/100 ml). ±S.D	† Mean size by AFM (nm) ±S.E
**Pit-M1**	349±1.4	-	-	-	-
**Pit-M2**	374±1.7	+	+	0.008±0.001	83.71±8.27
**Pit-M3**	364±1.5	+	-	-	-
**Pit-B4**	379±1.3	+	+	0.010±0.001	48.95±4.41
**Pit-P5**	373±2.6	+	-	-	-
**Pit-L6**	378±2.3	+	+	0.015±0.002	57.25±3.11
**Pit-L7**	371±2.8	+	+	0.007±0.001	151.78±9.10
**Pit-L8**	-	-	-	-	-
**Pit-O9**	377±1.7	+	+	0.005±0.002	135.77±6.10
**Pit-O10**	353±2.2	+	-	-	-
**Pit-O11**	-	-	-	-	-

There were highly significant differences in the quantity of milky ZnONps powders at alpha<0.01, which was higher in Pit-L6 than in Pit-M2, Pit-B4, Pit-L7, and Pit-O9 (Table 4).

**Table 4:** The statistical differences of the mean weight of ZnO nanopowders across *P. digitatum* isolates at alpha<0.01.

Weight of nanopowder Tukey HSD ^a^			
Strains	N	Subset for alpha=0.01	
		1	2
***P. italicum*-O9**	3	.0051	-
***P. italicum*-L7**	3	.0071	-
***P. italicum*-M2**	3	.0081	-
***P. italicum*-B4**	3	.0095	-
***P. italicum*-L6**	3	-	.0151
**Sig.**	-	.034	1.000

Means for groups in homogeneous subsets are presented. ^a^ – Uses Harmonic Mean sample size=3.000.

**Figure 4:**
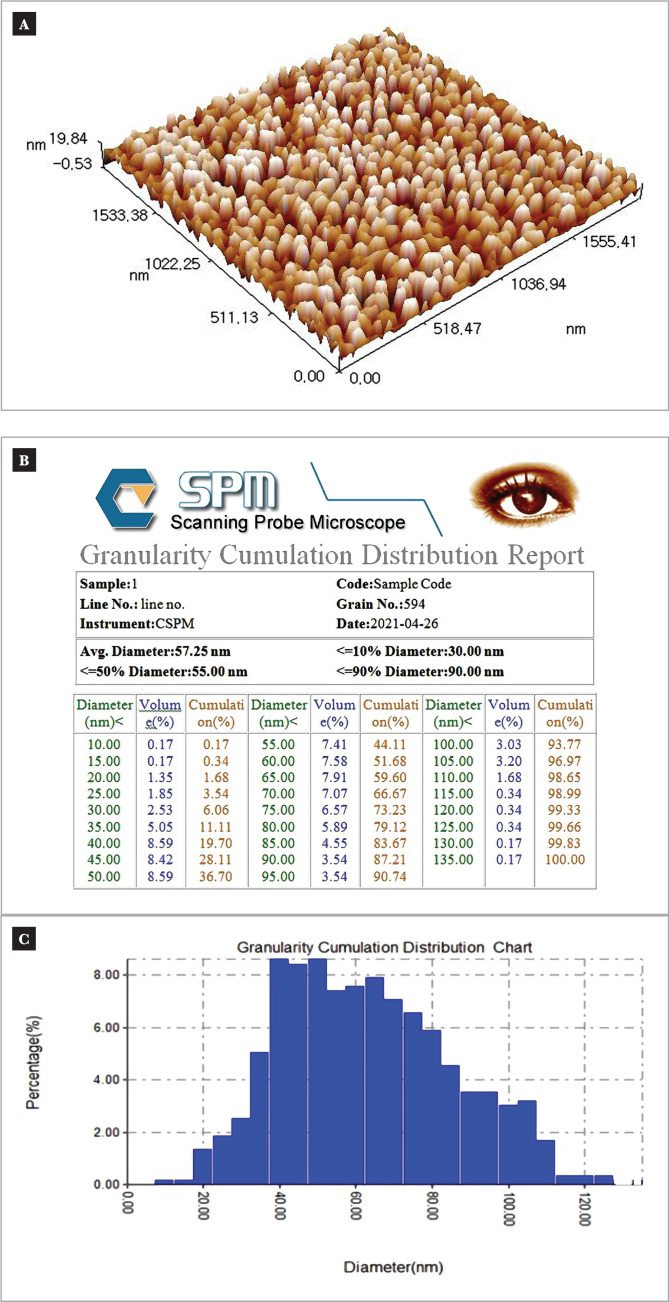
Surface roughness analysis, (B) distribution chart, and (C) distribution histogram, of ZnONPs manufactured by *Penicillin italicum* Pit-L6 (one of the triplicate test).

Also, regarding the mean size of nanoparticles shown in Table 5, there were no significant differences between Pit-B4 and Pit-L6 in the small average size of nanoparticles 48.95 nm and 57.25 nm, respectively, measured by AFM Figure 4 (A, B, and C), and both Pit-B4 and Pit-L6 isolates gave significantly smallest nanoparticles in mean diameter than the rest of the three isolates. Therefore they were selected as the best isolates for the bio-fabrication of ZnONps. However, because isolate Pit-L6 gave the highest amount significantly, it can be considered the most efficient and optimal isolate in manufacturing ZnONps nanoparticles.

**Table 5:** The statistical differences of average nanoparticle sizes by AFM amongst *P. digitatum* isolates capable of bio-fabrication of ZnO Nps at alpha<0.01.

Tukey HSD ^a^				
Strains	N	Subset for alpha=0.01		
		1	2	3
***P. italicum* B4**	3	48.9533	-	-
***P. italicum* L6**	3	57.2500	-	-
***P. italicum* M2**	3	-	83.7000	-
***P. italicum* O9**	3	-	-	135.7700
***P. italicum* L7**	3	-	-	151.7800
**Sig.**	-	.556	1.000	.079

Means for groups in homogeneous subsets are shown. ^a^ – Uses Harmonic Mean Sample Size=3.000.

### X-ray examination of FiBMs ZnONps

**Figure 5:**
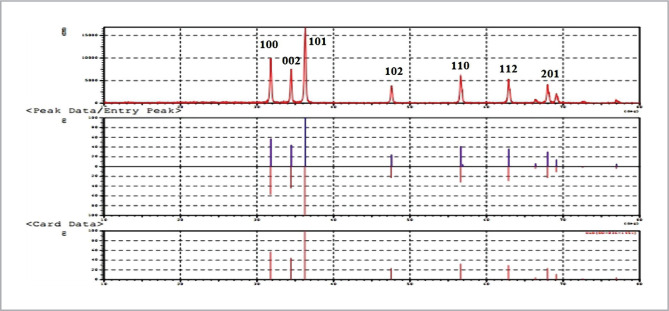
XRD pattern of biosynthesized ZnONps by extracellular free cells filtrate by *P. digitatum* Pit-L6.

X-ray diffraction was performed to substantiate various phases of ZnO nanoparticles for accurate determination of crystallographic characteristics and average size for ZnO nanoparticles of Pit-L6 isolate. The criterion wurtzite and XRD paradigms of ZnONps are depicted in Figure 5. Scherrer's formularization was used to calculate the average crystallite size of isolate Pit-L6 myco-ZnONps, which was 59.67 nm, close to AFM results.

### Micrograph image analysis of MycoZnONps by SEM and TEM

**Figure 6:**
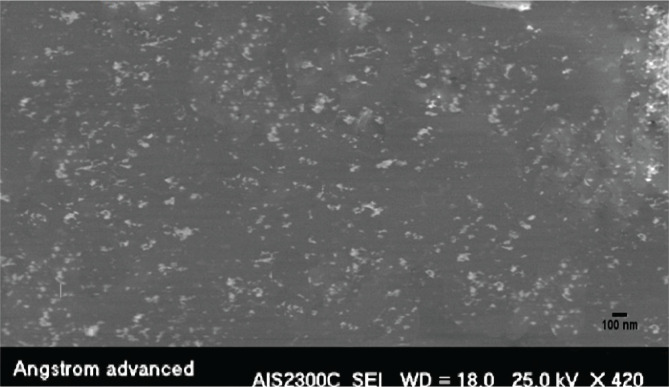
SEM image of ZnONps mycosynthesis by *P. italicum* Pit-L6.

**Figure 7:**
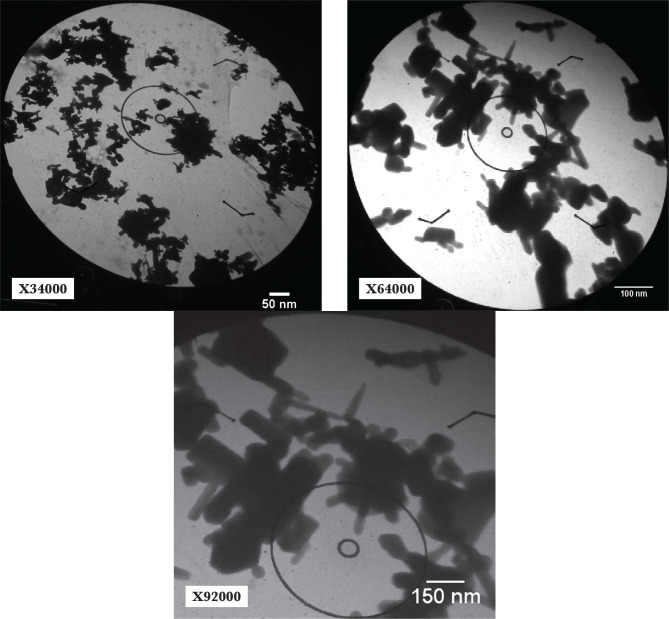
TEM images of different ZnO NPs shapes manufactured by *P. italicum* Pit-L6, X34000, X64000, and X92000.

**Figure 8:**
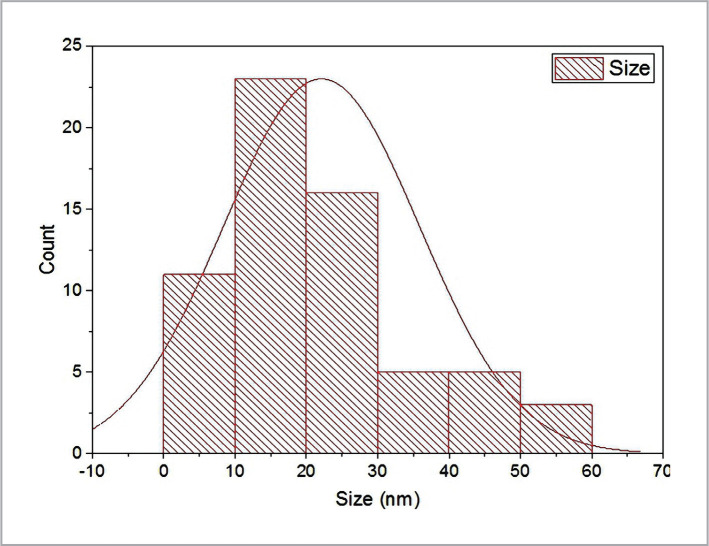
Histogram of average size accounted from TEM image of X64000.

**Figure 9:**
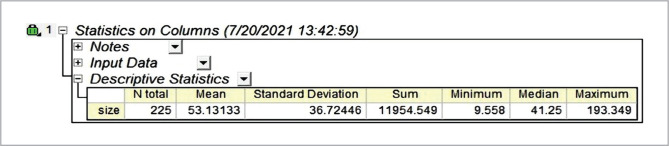
Table data of average size (53.13 nm) accounted from TEM image of X64000 of ZnONPs manufactured by *P. italicum* Pit-L6.

SEM and TEM micrographs were used to determine the conformation of the final result of the ZnONps morphology. Figure 6 is a micrograph of an SEM. On the other hand, TEM research revealed various geometrical crystalline structures in the morphology of ZnO nanoparticles, with an average size of 53.13 nm, a highly accurate result represented in Figures 7, 8, and 9. This resulted in smaller mean particle sizes than Rajan *et al*. (2016), who found the average ZnONps bio generation by filamentous *Aspergillus fumigatus* JCF at 60–75 nm, similar to the molds in this project [19].

## DISCUSSION

The diagnosis approach ignored other filamentous fungal species in favor of *P. italicum*, depending on characteristics such as the form and color of the fungus yarn, as well as the development velocity and area occupied by the colony in the culture dish over 96 hours on various culture medium, microscopic studies that involve staining with lactophenol cotton blue to reveal and quantify the precise structures (conidiophores, huge phialides, and conidia) as well as the cones of spores, their diameters, and forms of their surfaces and edges. All of these elements, as well as the genetic PCR method, are sufficient to meet the diagnostic aim required, consistent with other results [12, 17] where *Penicillium italicum* was isolated and identified from Iraqi citrus lemon and China citrus, respectively.

The formation of nebulous (hazy) filtrate with deposited white-milky precipitate was the initial detection step among FiGM isolates to select the strains capable of reducing zinc gluconohydraite to ZnONps. According to the recent study's findings, the wavelength of ZnONps biosynthesised by the yeast *Pichia fermentans* JA2 was 374 nm, agreeing with the suggestion of Chauhan *et al*. (2015) [24]. However, the wavelength peak of zinc nanoparticles generated by *Aspergillus terreus* in the Baskar *et al*. [15] search did not match the findings of this investigation, which was 340 nm. According to recent studies, differences in the diversity scope capability of ZnONps synthesis across fungal strains can be attributable to several variables: i- isolation sources, ii- micro creatures (fungi strains) diversity and adaptability growth, iii- substrate and nutrient utilization potential in bioreactor culture, iv- activation of biomolecules such as NADPH and NADH, and FAD which are involved in the biofabrication of metal nanoparticles [25, 26]

Because studying the surfaces of the materials' membranes is essential in understanding their distribution, the arrangement of atoms on surfaces, and identifying the homogeneity of properties, the atomic force microscope (AFM) was used to study the surface topography structure. Thus, it can study the effect of parameters on the properties of sedimentary material through microscopic analysis [21]. However, it should consider the amount (weight) of nanopowder (NPs) and the biosynthesis cost. In addition, it obtained an adequate small average size of nanoparticles within the dimensions of nanoscopy. Therefore, the Pit-L6 isolate significantly outperformed the rest of the isolates in terms of high quantity production and appropriate NPs small size, making it the preferable isolate for biomanufacturing of ZnONps in this study and ready for more ZnO nanoparticles characterization study. This result agreed with Issa *et al*. (2018) [10].

Furthermore, a particular software may be used to determine the roughness rate and granular size, as this measurement is more exact than X-ray analysis and provides a representation of the granular size distribution on the surfaces. X-ray powder diffraction (XRD) with two heat ranges of 20° to 80°, a scanning rate of 0.02° s-1, and a Cu K radiation of 0.1540 nm are used to identify the phase of ZnONps. The XRD peaks at 31.7°, 34.91°, 36.3°, 47.53°, 56. 61°, 67.96°, and 69.1° were identified as (1 0 0), (0 0 2), (1 0 1), (1 0 2), (1 1 0), (1 1 2), and (2 0 1), reflections, respectively. The sharper and stronger diffraction peaks show that the product has a fine crystalline structure, which is compatible with XRD peaks of ZnONps biosynthesis *Penicillium digitatum* Pdg-b3in the study of Issa (2022) [27].

The structural characterization of Nps was investigated using SEM to gain information on particle size, crystal structure, surface morphology, topography, and essential information of Nps at useful magnifications with virtually unlimited depth of field [1, 24]. Although these results differed somewhat from the X-ray diffraction and AFM analysis results, they remained within the nanoscale range. As a result, the final average size of the nanoparticle particles created by AFM, X-RD, SEM, and TEM is calculated using all of the data, the shape of plane faces, crystalloid diffractions, and topographical pictures received from AFM, X-RD, SEM, and TEM by *Penicillium italicum* Pit-L6 depending on current growth and biofabrication circumstances. The range will be around 53.25 nm to 59.67 nm. Our findings contradicted those of Shamsuzzaman *et al*. (2013), who found that the average size of ZnONps created by *Candidia Albicans* by SEM and TEM was 15–20 nm and 20 nm, respectively [28]. This might be due to variances in the kinds of microorganisms and substrates used. The ZnONps in this study generated by enzymatical reduction of *P. italicum* Pit-6 filtrate with these characteristics can be used as antimicrobial agents against virulent solid and multidrug-resistant pathogenic bacteria like Pseudomonas aeruginosa [29] and Salmonella-typhi [30].

## CONCLUSION

In terms of substance, the current study was the first regional investigation on the utilization of *Penicillium italicum* in the biosynthesis of ZnO nanoparticles, also concluding that the extracellular reduction by free-cell filtrate of *P. italicum* Pit-L6 was the best among other FiGM strains isolated from rotten citrus fruits with an extraordinarily low pH, as well as a simple methodology for fabricating ZnO nanoparticles by reducing Zincum Gluconicum hydrate, with an average size of 53.25 nm to 69.67 nm. It was characterized by various complimentary mechanization and gadgets in diverse combinations, including UV-visible, AFM, X-RD, SEM, and TEM. These results depended on the kind of species strain, substrate utilized, liquid culture, fermentation conditions, and characterization devices. The enhanced characterization via zeta-potential, LDS, and FTIR are perfect for complete analyses. Also, the *in vitro* and *in vivo* experiments as antimicrobial agents benefit the boosting applications. The method enables the biofabrication of well-structured shaped ZnO nanoparticles in an environmentally friendly, simple to scale–up, inexpensive, and efficient manner, resulting in innovative and safe resources for a wide range of human applications. Furthermore, it opens the opportunity for future studies on this optimum strain or other fungal genus and species in the biosynthesis of other metal oxide nanoparticles and their applications in various biotechnology, biomedical, nutritious food, agricultural, and industrial fields.

## Personal thanks

The authors would like to express appreciation to the University of Thi-Qar, College of Science and College of Medicine, Al-Nahrani University/Iraq, and Central Fungi Laboratory of Postgraduate - Faculty of Science, the University of Basra for their support and help in completing the project.

## References

[1] Yusof HM, Mohamad R, Zaidan UH, Nor' Aini Abdul Rahman NA (2019). Microbial synthesis of zinc oxide nanoparticles and their potential application as an antimicrobial agent and a feed supplement in the animal industry: a review. J. of Animal Sci. and Biotech.

[2] Li H, Yan S, Deng C, Dou S, Ren X (2021). Synthesis and characterization of Ag nanoflowers with different morphologies. IOP Conf Series.: Mater. Scie. and Engin.

[3] Kareem S, Altimimi MB, Jarullah BA (2017). Improvement of Newcastle disease virus vaccine by using gold nanoparticles and some natural food additives. Uni. Thi-Qar. J. Sci.

[4] Tuhmaz HH (2020). The thermo-optical treatment of cancer by laser light with Ag nanoparticles. University of Thi-Qar Journal of Science.

[5] da Silva BL, Abuçafy MP, Manaia EB, Junior JAO (2019). Relationship Between Structure And Antimicrobial Activity Of Zinc Oxide Nanoparticles: An Overview. Inter. J. of Nanomedicine.

[6] Alavi M, Nokhodchi A (2020). An overview d antimicrobial and wound healing properties of ZnO nanobiofilms, hydrogels, and bionanocomposites based on cellulose, chitosan, and alginate polymers. Carbohydrate Polymers.

[7] Alavi M, Nokhodchi A (2021). Synthesis and modification of bio-derived antibacterial Ag and ZnO nanoparticles by plants, fungi, and bacteria(review). Drug Discovery Today.

[8] Kalpanaa VN, Katarua BAS, Sravania N, Vigneshwaria T (2018). Biosynthesis of zinc oxide nanoparticles using culture filtrates 0f Aspergillus niger: Antimicrobial textiles and dye degradation studies. OpenNano.

[9] Hefny ME, El-Zamek FI, Abd El-Fattah HI, Mahgoub SAM (2019). Biosynthesis of zinc nanoparticles using culture filtrates of *Aspergillus, Fusarium* and Penicillium fungal species and their antibacterial properties against gram-positive and gram-negative bacteria. Biotech. Res.

[10] Issa MAS, Al-Sheikhly AART, Hamid MK, Nader MI (2018). Novel rapid green fabrication of ZnONps using mycofiltrate by local fungus *Aspergillus Parasiticus* Ap4. Biosc. Res.

[11] Housseiny MM, Gomaa EZ (2019). Enhancement of Antimicrobial and Antitumor Activities of Zinc Nanoparticles Biosynthesized by *Penicillium chrysogenum* AUMC 10608 Using Gamma Radiation. Egypt. J. Bot.

[12] Taha ZK, Howar SN, Sulaiman GM (2019). Isolation and Identification of *Penicillium italicum* from Iraqi Citrus Lemon Fruits and its Ability Manufacture of Silver Nanoparticles and their Antibacterial and Antifungal activity. Resea. J. Pharm. and Tech.

[13] Elfita E, Muharni M, Yohandini H, Fadhillah F (2021). Antioxidant activity of endophytic fungi isolated from the stem bark of Swietenia mahagoni (L.). Jacq. IOP Conf. Series.: Mater. Sci. and Engin.

[14] Sri VS, Rajagopal K (2016). Isolation and Identification of Thermo Tolerant Endophytic Fungi from Melia dubia and Synthesis of Zinc Nano Particles. Intern. J. of NanoSci. and Nanotech.

[15] Pitt JI, Hocking DA (2009). Fungi and Food Spoilage. Springer Dordrecht Heidelberg London and New York Book.

[16] Visagie CM, Houbraken J, Frisvad JC, Hong SB (2014). Identification and nomenclature of the genus Penicillium. Studies in Mycology.

[17] Chen K, Tian Z, Jiang F, Chao-an Long C (2019). Development of *Penicillium italicum* Specific Primers for Rapid Detection among Fungal Isolates in Citrus. J. Microbiol. Biotechnol.

[18] Ottoni CA, Simões MF, Fernandes S, dos Santos JG, da Silva ES, de Souza RFB, Maiorano AE (2017). Screening of filamentous fungi for antimicrobial silver nanoparticles synthesis. AMB Express.

[19] Rajan A, Cherian E, Baskar G (2016). Biosynthesis of zinc oxide nanoparticles using *Aspergillus fumigatus* JCF and its antibacterial activity. Interna. J. of Modern Scie. and Techno.

[20] Baskar G, Chandhuru J, Fahad KS, Praveen AS (2013). Mycological Synthesis, Characterization and Antifungal Activity of Zinc Oxide Nanoparticles. Asian Pharma Press.

[21] Mahmood S, Mandal UK (2017). Morphological Characterization of Lipid Structured Nanoparticles by Atomic Force Microscopy while Minimizing the Formation of Failed Artefacts. Current Nanomaterials.

[22] Bayroodi E, Jalal R (2016). Modulation of antibiotic resistance in Pseudomonas aeruginosa by ZnO nanoparticles. Iranian J. of microbio.

[23] Al-zamily RTN, Al-assadi AKG, Issa MAS (2016). Production of single cell oil by local isolate of mucor species using by-products as carbon and nitrogen sources and determination of fatty acids profile. International Journal of Agricultural Science and Research.

[24] Chauhan R, Reddy A, Abraham J (2015). Biosynthesis of silver and zinc oxide nanoparticles using *Pichia fermentans* JA2 and their antimicrobial property. Der Pharma Chemica.

[25] Singh P, Kim YJ, Zhang D, Yang DC (2016). Biological Synthesis of Nanoparticles from Plants and Microorganisms. Trends in Biotech.

[26] Khandel Pramila A, Shahi K (2016). Microbes mediated synthesis of metal nanoparticles: current status and future prospects. Int. J. of Nanomaterials and Biostructures.

[27] Issa MAS (2022). Rapid enzymatical reduction of zincum gluconicum for biomanufacturing of ZnO nanoparticles by myco extracellular filtrate of *Penicillium digitatum* Pdig-B3:as a soft green technique. Archives of Razi Institute.

[28] Shamsuzzaman Mashrai A, Khanam H, Aljawf RN (2013). Biological synthesis of ZnO nanoparticles using C albicans and studying their catalytic performance in the synthesis of steroidal pyrazolines. Arabian Journal of Chemistry.

[29] Nader MI, Kareem AAA, Rasheed MN, Issa MAS (2017). Biofilm Formation and Detection of pslÁ Gene in Multidrug Resistant Pseudomonas aeruginosa Isolated from Thi-Qar, Iraq. Iraqi Journal of Biotechnology.

[30] Hanan ZK, Saleh MB, Mezal EH, Issa MAS (2021). Phylogenitic analysis of Biofilm Association Protein (BapA) amplicons in Salmonella Typhi Carrier in Gallbladder Diseases Patients in Thi-Qar Province/Iraq. International Journal of Aquatic Science.

